# Enhancing chemical synthesis: a two-stage deep neural network for predicting feasible reaction conditions

**DOI:** 10.1186/s13321-024-00805-4

**Published:** 2024-01-24

**Authors:** Lung-Yi Chen, Yi-Pei Li

**Affiliations:** 1 Department of Chemical Engineering, National Taiwan University, No. 1, Sec. 4, Roosevelt Road, Taipei, 10617 Taiwan; 2 Taiwan International Graduate Program on Sustainable Chemical Science and Technology (TIGP-SCST), No. 128, Sec. 2, Academia Road, Taipei, 11529 Taiwan

**Keywords:** Reaction condition, Recommendation system, Multi-task modeling, Multi-label classification

## Abstract

**Abstract:**

In the field of chemical synthesis planning, the accurate recommendation of reaction conditions is essential for achieving successful outcomes. This work introduces an innovative deep learning approach designed to address the complex task of predicting appropriate reagents, solvents, and reaction temperatures for chemical reactions. Our proposed methodology combines a multi-label classification model with a ranking model to offer tailored reaction condition recommendations based on relevance scores derived from anticipated product yields. To tackle the challenge of limited data for unfavorable reaction contexts, we employed the technique of hard negative sampling to generate reaction conditions that might be mistakenly classified as suitable, forcing the model to refine its decision boundaries, especially in challenging cases. Our developed model excels in proposing conditions where an exact match to the recorded solvents and reagents is found within the top-10 predictions 73% of the time. It also predicts temperatures within ± 20 °**C** of the recorded temperature in 89% of test cases. Notably, the model demonstrates its capacity to recommend multiple viable reaction conditions, with accuracy varying based on the availability of condition records associated with each reaction. What sets this model apart is its ability to suggest alternative reaction conditions beyond the constraints of the dataset. This underscores its potential to inspire innovative approaches in chemical research, presenting a compelling opportunity for advancing chemical synthesis planning and elevating the field of reaction engineering.

**Scientific contribution:**

The combination of multi-label classification and ranking models provides tailored recommendations for reaction conditions based on the reaction yields. A novel approach is presented to address the issue of data scarcity in negative reaction conditions through data augmentation.

**Graphical Abstract:**

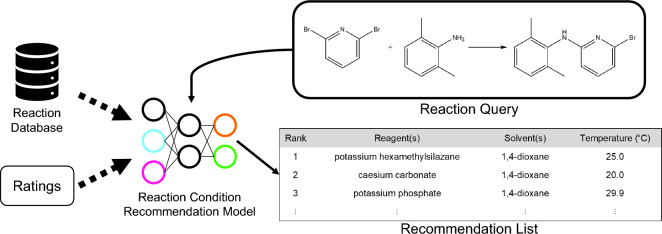

**Supplementary Information:**

The online version contains supplementary material available at 10.1186/s13321-024-00805-4.

## Introduction

In recent years, computer-aided synthesis planning (CASP) [1–4] has emerged as an automatic approach for designing synthesis routes of new chemicals [5, 6]. This development has been facilitated by extensive research on predicting retrosynthesis steps [7–10] and the algorithms that guide machines in finding the most suitable synthetic pathways [11–13]. However, an important consideration when carrying out actual synthesis in the laboratory is the selection of proper reaction conditions to maximize yields for each reaction. This consideration is crucial for reducing the cost of purification and maximizing the overall yield of the synthesis route leading to the final product [14–16]. By suggesting suitable reaction conditions such as reagent, solvent, and catalyst, CASP can help researchers save time and resources in their quest for more efficient and effective reactions. This capacity opens up new possibilities for accelerating the discovery and development of chemical compounds, ultimately contributing to advancements in various fields such as pharmaceuticals, materials science, and sustainable chemistry.

Nevertheless, predicting reaction conditions is a challenging task due to the complicated interactions between the chemicals involved. The compatibility between the reagent and solvent is crucial for a successful reaction, as they should not react and generate unwanted byproducts that impede the desired outcome. Furthermore, the existence of multiple combinations of reaction conditions that can achieve the desired result further complicates the task of recommending precise reaction contexts. As a result, researchers traditionally rely on empirical knowledge, experience, and heuristic approaches to identify a suitable set of conditions [17, 18].

Over the past few years, the field of chemistry has witnessed the widespread application of machine learning in various areas, including molecular property prediction [19–22], drug and material design [23–26], and chemical biology [27–30]. Additionally, machine learning has proven valuable in navigating the vast parameter space of chemical reactions and proposing promising reaction conditions [31]. For example, Gao et al. [32] introduced a neural network architecture with features resembling a recurrent neural network. This model achieved high accuracy by sequentially predicting catalysts, solvents, reagents, and temperatures, taking into account their interdependence across a broad spectrum of organic reactions. Maser et al. [33] focused on elucidating the roles of different species involved in reactions, such as metals, ligands, bases, solvents, and additives. They tackled the prediction of reaction conditions by developing multi-label classification models specifically tailored for Suzuki, Negishi, C-N couplings, and Pauson-Khand reactions. Recently, Kwon et al. [34] pointed out that previous techniques provided only a single prediction per reaction, lacking a comprehensive list of possible reaction conditions. To overcome this issue, they developed a generative variational autoencoder that predicted multiple reaction conditions through repeated sampling from the output distribution. The resulting list of sampled conditions could then be evaluated by human experts or another model to rank their feasibility.

This study introduces a model capable of predicting various combinations of reaction conditions suitable for a given reaction and ranking them based on the expected product yield. The model design is inspired by the two-stage recommendation systems commonly used in online shopping [35, 36], and video recommendation [37, 38]. For example, Covington et al. [37] employed a similar strategy by using a candidate generation model with user features as inputs to identify relevant videos in the corpus, followed by a ranking model in the second stage to assign scores exclusively to those relevant items. This approach is particularly effective when dealing with large search spaces. By efficiently identifying relevant items, the candidate generation model significantly reduces the search space, thereby reducing computation time during the ranking stage. Considering the immense number of possible combinations of reaction conditions, we developed a similar two-stage model to recommend and rank feasible reaction conditions based on their respective yields. The first part of the model generates a variety of potential reagents and solvents for a reaction, while the second part predicts temperatures and ranks the conditions using relevance scores calculated from the anticipated product yield. The model was trained on a diverse dataset encompassing ten reaction types, including Buchwald-Hartwig cross coupling, Chan-Lam coupling, Diels-Alder, Fischer indole synthesis, Friedel-Crafts acylation, Friedel-Crafts alkylation, Grignard reaction, Kumada coupling, Negishi coupling, and reductive amination. Overall, this work contributes to the advancement of CASP by addressing the prediction of multiple combinations of reaction conditions, providing a more comprehensive and systematic approach to optimizing reactions.

## Method

### Data preparation and preprocessing

**Fig. 1:**
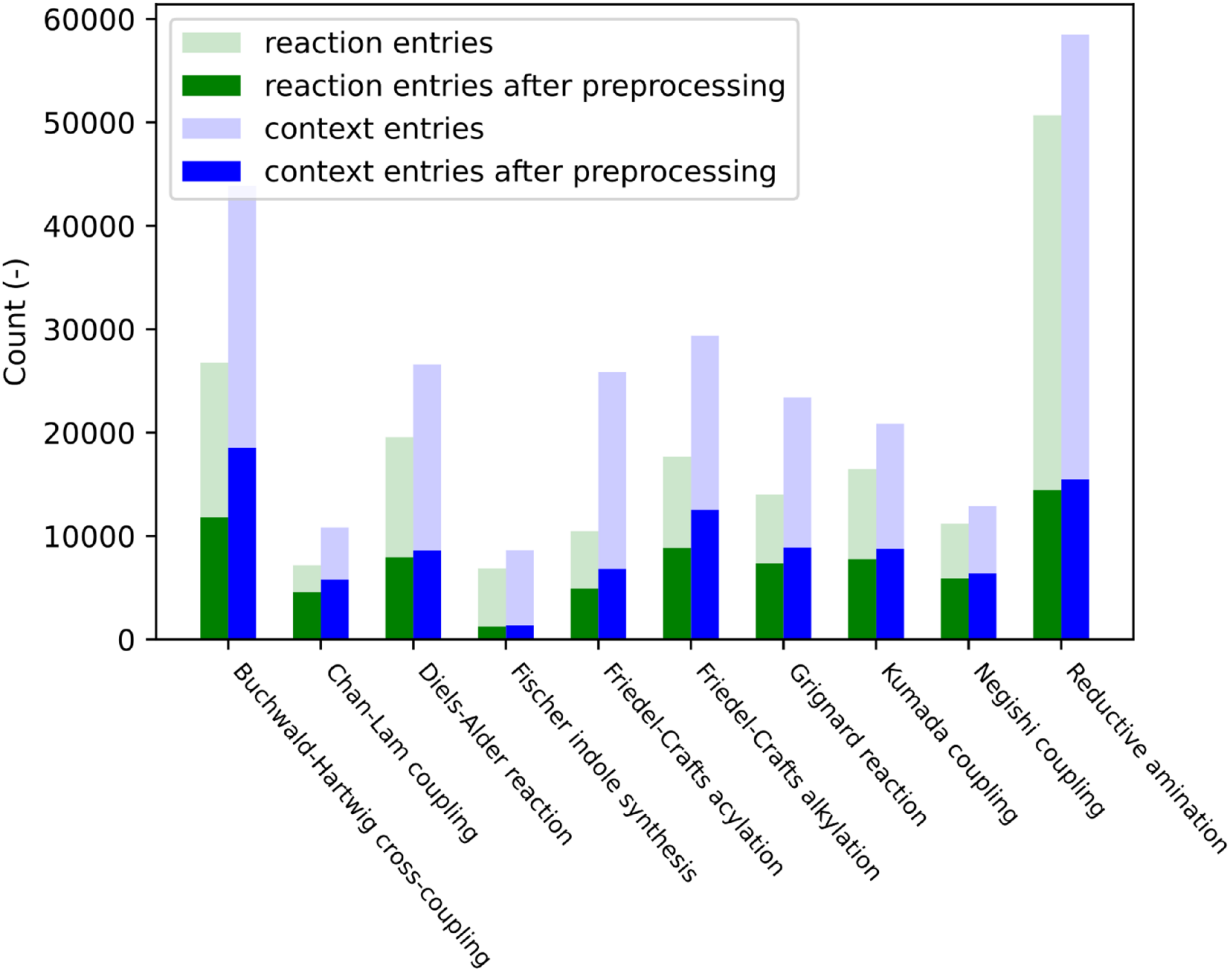
Data distribution across different reaction types. Translucent and solid bars represent the number of data points before and after preprocessing, respectively

The reaction datasets used in this study were obtained from Reaxys [39], and their distribution across various reaction types is shown in Fig. 1. In Reaxys, chemicals that facilitate reactions are categorized as solvents, reagents, or catalysts. However, the obtained datasets contained a limited number of records (1.57%) that specifically mentioned catalysts, as most metal catalysts were primarily categorized as reagents. To eliminate ambiguity arising from chemical categorization, we merged the reagent and catalyst categories, collectively designating these chemicals as reagents. Additionally, we observed instances where certain chemicals appeared in both the reagent and solvent categories in Reaxys. For example, while methanol is predominantly considered a solvent in most reaction entries, there are a few cases where it is categorized as a reagent. To address this issue, we redefined the role of each chemical based on the category in which it appeared most frequently. This approach reduces the likelihood of the model predicting the same chemicals for both solvent and reagent tasks, leading to more accurate predictions.

Inconsistencies in naming the same chemical species posed another challenge in the dataset. To address this, we used OPSIN [40], PubChem [41], and ChemSpider [42] to obtain canonicalized SMILES representations of the chemical names and merged them if they shared identical SMILES representations. We note that this work used the anhydrous form of SMILES to represent chemicals. For instance, sodium carbonate monohydrate and sodium carbonate were considered the same reagent. Furthermore, some reactions in the dataset involved an unusually high number of solvents and reagents. To maintain focus and simplify the analysis, such rare cases, which exceeded two solvents and three reagents per reaction entry, were excluded from the study. This constraint led to the removal of approximately 5.33% of the data from the analysis. The completed data preprocessing workflow is outlined below:

1. Removal of data with reaction SMILES that cannot be parsed by RDKit [43].
2. Removal of data without solvent and yield records.
3. Removal of data with reaction conditions that involve more than two solvents or three reagents.
4. Reassignment of the category label of a chemical to either solvent or reagent based on the category in which the chemical appeared most frequently.
5. Removal of entries with rare reagents and solvents whose frequency in the dataset is less than 10.
6. Standardization of labels by using OPSIN [40], PubChem [41], ChemSpider [42] to obtain the SMILES representation of chemical names. Labels with identical SMILES were merged, while labels without corresponding SMILES were kept unchanged.
7. Random splitting of the dataset into training, validation, and testing sets with an 8:1:1 ratio. Reaction entries with the same reaction SMILES but with different reaction conditions were assigned to the same subset, ensuring that no learned reaction appears during validation and testing.

Following the preprocessing steps, the remaining dataset consists of 74,683 reaction entries and 93,081 reaction conditions. There are 1320 labels for the reagent class and 87 labels for the solvent class.

### Model setup

**Fig. 2:**
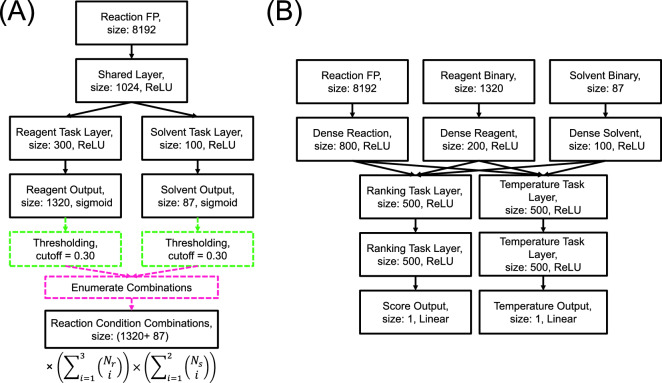
The architecture of the reaction context recommendation model. **A** The initial component is the candidate generation model, comprising a feedforward neural network. This model encodes reaction fingerprints and predicts the probabilities of the solvents and reagents that might be relevant to the reaction as a multi-label classification problem. The predicted relevant solvents and reagents are then enumerated combinatorically to generate a list of possible reaction contexts for the reaction. **B** Subsequently, the ranking model predicts the temperature and relevance score for each generated reaction context from the first model

As shown in Fig. 2, the reaction context recommendation system proposed in this study comprises two consecutive prediction stages: candidate generation and candidate ranking. The model predicts a subset of potential reagents and solvents in the initial candidate generation stage based on the given reaction query. This particular subset is then utilized in the subsequent stage to enhance the accuracy of the ranking model by excluding irreverent reagents and solvents.

The candidate generation model was implemented using a multi-task neural network designed for multi-label classification, which includes a shared hidden layer followed by two task-specific output layers, as shown in Fig. 2A. To effectively capture relevant chemical information from the provided reaction query, a reaction fingerprint was employed as input. This reaction fingerprint [32, 44] was generated by concatenating two distinct components: the Morgan circular [45] fingerprint (with a radius of 2 and a length of 4096) of the product, and the disparity between the fingerprints of the reactant and the product. As shown in Fig. 2A, the candidate generation model has two separate output layers, which are responsible for predicting solvent labels (with a length of 87) and reagent labels (with a length of 1320), respectively. The prediction losses for reagent and solvent outputs were calculated using a focal loss function [46]

1 $$\begin{aligned} \text {Focal Loss}(p,y)= {\left\{ \begin{array}{ll} -(1-p)^{\gamma }{{\log }(p)}, &{} \text {if } y = 1; \\ -p^{\gamma }{{\log }(1-p)}, &{} \text {otherwise}. \end{array}\right. } \end{aligned}$$

where $\gamma \ge 0$ is a modulating factor that concentrates training on misclassified hard examples, $p\in [0,1]$ is the predicted probability from the model, and $y\in \left\{ 0,1\right\}$ is the binary indicator if the reagent or solvent label is the correct classification for the reaction. The selection of the focal loss function stems from its efficacy in addressing class imbalance issues (as shown in Additional file 1: Figure S1) and giving more weight to misclassified instances [47, 48]. The modulating factor $\gamma$ is a hyperparameter that was manually adjusted for better performance of the model (see Additional file 1: Table S1). In the candidate generation model, the losses for the reagent ($\mathcal {L}_r$) and solvent ($\mathcal {L}_s$) prediction tasks were combined using the homoscedastic uncertainty approach [49]

2 $$\begin{aligned} \mathcal {L}={\frac{1}{{2\sigma }_r}\mathcal {L}_r}+ {\frac{1}{{2\sigma }_s}\mathcal {L}_s}+{\log }{\sigma _r\sigma _s} \end{aligned}$$

where $\mathcal {L}$ is the cumulative loss, and $\sigma _r$ and $\sigma _s$ are the homoscedastic uncertainties of the reagent and solvent prediction tasks learned by the model during training. These uncertainties can be viewed as automatically adjusted weights between the losses of the two prediction tasks. To identify potential candidates for solvents and reagents in the given reaction, we considered predicted probabilities of labels exceeding a certain threshold. In this work, a threshold of 0.3 was selected due to its optimal performance in validation, as will be discussed in the subsection below. The shortlisted candidates for solvents and reagents were then subjected to a combinatorial enumeration process, generating all possible combinations of reaction conditions derived from these solvent and reagent candidates. The total count of generated reaction contexts can be computed as

3 $$\begin{aligned} \left( \sum _{i=1}^{3} \left( {\begin{array}{c}N_r\\ i\end{array}}\right) \right) \times \left( \sum _{i=1}^{2}\left( {\begin{array}{c}N_s\\ i\end{array}}\right) \right) \end{aligned}$$

where $N_r$ and $N_s$ are the numbers of reagents and solvents with probabilities higher than the threshold, and $$\left( {\begin{array}{c}.\\ .\end{array}}\right) $$ is the binomial coefficient.

The candidate generation model serves as an initial filter, identifying potential reagents and solvents, and generating various reaction contexts based on the selected reagents and solvents for a reaction. As shown in Fig. 2B, a separate model evaluates and ranks these reaction contexts. In this study, we employed a listwise approach similar to ListNet [50] for the purpose of ranking the reaction contexts. To implement this approach, we began by assigning a relevance score (*s*) to each set of reaction conditions. These relevance scores are arbitrary numerical values where larger values indicate better suitability of the conditions for the given reaction. The definition of these relevance scores can take into account factors such as reagent and solvent costs, reaction temperature, and separation feasibility, depending on user objectives. For simplicity, we calculated relevance scores using the product yield $(s=2\times yield+2)$ to prioritize reaction conditions that promote the formation of the target product. If a reaction context involves a reagent or solvent that is absent from the actual reaction data, a relevance score of 0 is assigned. For a collection of *n* reaction contexts, the probability of a particular context being ranked as the top one can be computed using the following formula [50]

4 $$\begin{aligned} P_s(i)=\frac{e^{s_i}}{\sum _{i=1}^{n}e^{s_i}} \end{aligned}$$

where $s_i$ is the relevance score of the *i*-th condition combination. Figure 2B illustrates the architecture of the ranking model, which takes the reaction fingerprint and one-hot encoded vectors for the solvent and reagent as inputs. These inputs pass through separate dense layers and then combine to form a concatenated representation, which proceeds through two specialized layers: one for ranking reaction conditions and the other for temperature prediction. In this work, the loss for temperature prediction was computed using the mean square error, whereas the ranking loss was calculated using the Kullback–Leibler divergence between the predicted probability and the probability calculated using relevance scores derived from yield. The losses from both tasks were merged and weighted using the same homoscedastic uncertainty method described in Eq. 2.

### Data augmentation by hard negative sampling

In data-driven chemistry, the inclusion of negative data, such as non-reactive and non-active molecular structures, is crucial for effective model learning [51]. A prevalent issue in chemical reaction databases derived from literature is the scarcity of low-yield reaction samples. This gap significantly hinders the development of accurate and comprehensive predictive models. Previous work has demonstrated the necessity for documentation of all data pertaining to new chemical reactions [52]. Such detailed record-keeping is key to enhancing the quality of reaction databases, which, in turn, improves the models trained on these databases. Furthermore, Tripp et al. highlighted a critical challenge in training retrosynthesis models [53]. They note that models trained solely on positive data may erroneously generate unrealistic reaction pathways. To address this issue, Tripp et al. proposed the development of a reaction synthesis probability assessment model. This model aims to mitigate the risk of incorrect outcomes by factoring in the likelihood of a reaction’s success.

**Fig. 3:**
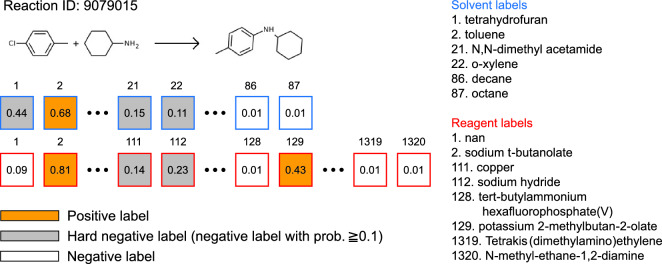
An illustration of negative sampling. The numbers in the blue and red squares represent the predicted probabilities assigned to each solvent and reagent label, respectively. Chemicals recorded in the reaction conditions in Reaxys are marked as positive labels (shown in orange). On the other hand, reagents and solvents not utilized in the actual reaction data but having predicted probabilities surpassing 0.1 are classified as hard negative labels (depicted in gray). This indicates that these chemicals are not actually pertinent to the reaction, yet the model might mistakenly consider them as feasible solvents or reagents

Similarly, the challenge of needing both positive and negative data is observed in the training of ranking models [54]. This further emphasizes the need for a balanced approach to data collection and model training in the field of data-driven chemistry. To overcome this limitation, the technique of negative sampling [55–58] was employed. As illustrated in Fig. 3, this approach involved generating additional negative data by identifying reagents and solvents that were not present in actual reaction data but were predicted by the candidate generation model to have a probability exceeding 0.1. These instances, referred to as “hard negative labels,” represented irrelevant reagent or solvent candidates that the model might mistakenly consider as suitable.

**Fig. 4:**
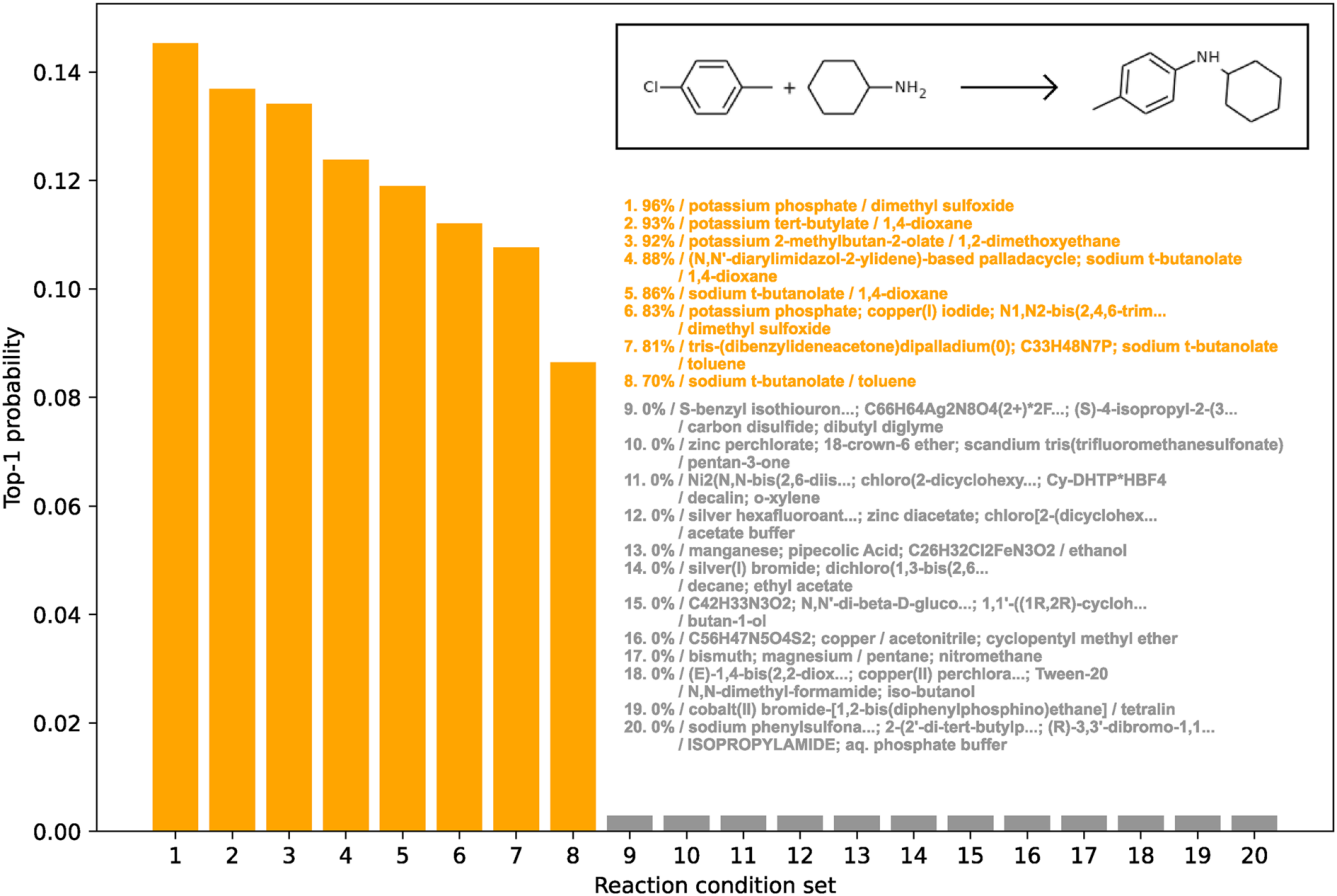
An illustration of the ranking of recorded reaction conditions (orange) and sampled hard negative reaction conditions (gray)

To enrich the training process, both positive labels and hard negative labels were combined and subjected to combinatorial enumeration, resulting in a wide range of both suitable and unsuitable combinations of reaction conditions as illustrated in Fig. 4. Combinations with hard negative labels were assigned a relevance score of 0. This approach, known as hard negative sampling [59, 60], enhanced the training of the ranking model by exposing it to a more diverse set of reaction contexts. This, in turn, improved the model’s ability to differentiate between suitable and unsuitable reaction conditions, particularly in challenging cases.

### Evaluation metrics

The candidate generation model in this study predicts potential reagents and solvents using a multi-label prediction framework, which can result in fully correct, partially correct, or fully incorrect predictions. Suppose $x\in \mathbb {R}^d$ denotes the *d*-dimensional instance space, and $Y\in 2^q$ denotes the label space with *q* defined labels in the class. Given the test set $S=\left\{ (x_i,Y_i))|1 \le i \le N_{test}\right\}$, to evaluate the performance of the multi-label predictions $Z\in 2^q$ derived from the candidate generation model, we adopted the hamming loss and the example-based evaluation metrics specific to multi-label learning, which are defined as [61]

5 $$\begin{aligned} \text {Hamming Loss}= & {} \frac{1}{N_{test}}\sum _{i=1}^{N_{test}}\frac{1}{q}|Z_i\Delta Y_i|\end{aligned}$$

6 $$\begin{aligned} \text {Precision}= & {} \frac{1}{N_{test}}\sum _{i=1}^{N_{test}}\frac{|Y_i\cap Z_i|}{|Z_i|}\end{aligned}$$

7 $$\begin{aligned} \text {Recall}= & {} \frac{1}{N_{test}}\sum _{i=1}^{N_{test}}\frac{|Y_i\cap Z_i|}{|Y_i|}\end{aligned}$$

8 $$\begin{aligned} \text {F1-score}= & {} \frac{1}{N_{test}}\sum _{i=1}^{N_{test}}\frac{2|Y_i\cap Z_i|}{|Y_i|+|Z_i|}=2\times \frac{\text {Precision}\times \text {Recall}}{\text {Precision}+\text {Recall}} \end{aligned}$$

where $\Delta$ stands for the quantity of differences between the predicted label and the true label. Hamming loss is commonly used to measure differences between predicted and true labels. However, this metric itself may not be adequate for evaluating the candidate generation model because many reactions involve only a few solvents and reagents. Even a model that always predicts zero probability for all labels can achieve a low hamming loss in such cases. To provide a more meaningful evaluation, precision assesses the reliability of positive predictions, while recall evaluates the model’s ability to capture positive labels. The F1-score, a balanced metric that combines precision and recall, offers a comprehensive assessment of the candidate generation model’s performance, particularly in scenarios where reactions involve a limited number of solvents and reagents. These metrics are essential for evaluating the model’s effectiveness accurately.

## Results and discussion

### Threshold optimization for candidate generation model

**Fig. 5:**
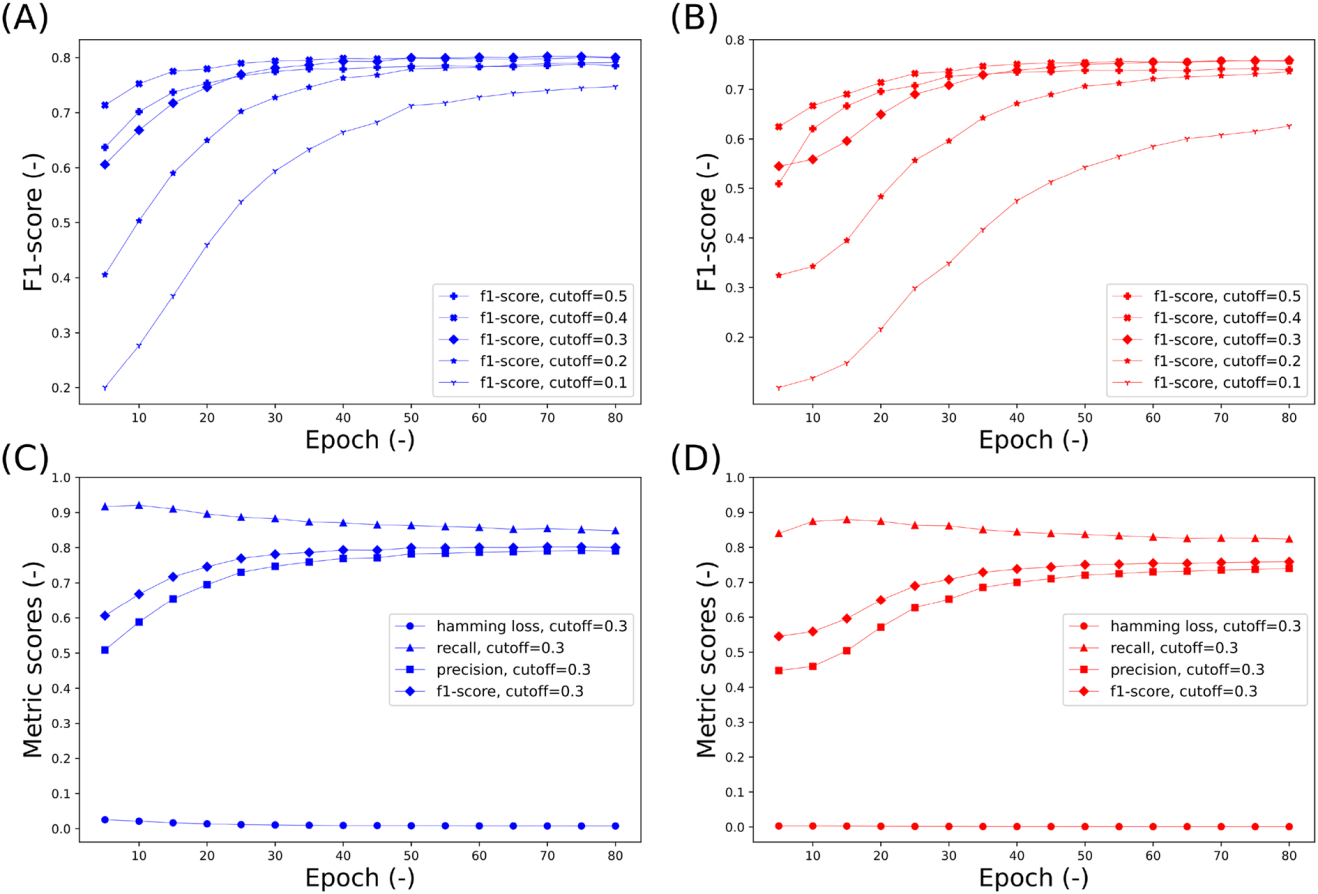
Validation evaluation metrics for solvent (blue) and reagent (red) multi-label classification. Panels **A** and **B** depict the evolution of F1-scores across epochs using various cutoffs. A cutoff value of 0.3 yields the highest F1-score in the concluding epochs, leading to the adoption of this threshold for categorizing labels as positive or negative in the candidate generation model. Panels **C** and **D** showcase hamming loss and example-based precision, recall, and f1-score, all calculated using a 0.3 cutoff

In the candidate generation model, labels with predicted probabilities surpassing a designated threshold were considered as potential solvents and reagents for a given reaction. The threshold value for this selection was determined using the highest F1-scores observed during the validation process. Figure 5 illustrates the gradual increase and eventual plateau of the F1-score on the validation set as the training epochs progress. Notably, a threshold of 0.3 yielded the highest F1-score during the last several epochs in validation. As a result, chemicals with predicted probabilities exceeding 0.3 were chosen as candidate solvents or reagents for the specified reaction in this study.

The hamming loss, precision, recall, and F1-score at the threshold of 0.3 are shown in Figs. 5C and D. As previously discussed, because a significant number of reaction instances involve only a small subset of the solvents and reagents in the list, the candidate generation model can achieve low hamming loss by assigning low probabilities to all solvent and reagent labels. Therefore, relying solely on hamming loss for evaluating the effectiveness of the candidate generation model is insufficient.

It is important to note that increasing the threshold reduces the number of predicted feasible reagents and solvents, resulting in a more limited list of recommended reaction conditions. Conversely, decreasing the cutoff enhances the recall score by encompassing more labels with slightly lower probabilities as positive classifications. Nevertheless, setting the cutoff too low extends the list of recommended reaction contexts, posing challenges for the subsequent ranking model when sorting predictions. Therefore, selecting an appropriate threshold value is crucial to ensure that the candidate generation model functions effectively as an initial filter for identifying potential reagents and solvents.

### Performance of the two-stage model

While the two-stage model can propose multiple reaction conditions for each reaction, our initial assessment focused on its ability to accurately predict at least one reaction condition for each reaction within the test set. Success in this evaluation was defined strictly as achieving an exact match with the reaction conditions as they appear in the dataset, and the results can be found in Table 1. This criterion is stringent because there are cases where, for instance, a solvent could be replaced by another solvent with similar properties. In certain instances, publications might suggest possible solvent or reagent substitutions for a reaction. However, the database typically documents only a single condition, often the one yielding the highest yield. As a result, assessing the validity of proposed conditions that partially match the recorded ones becomes challenging due to the lack of comprehensive data. Therefore, our evaluation exclusively considered exact matches. Nonetheless, the model successfully predicted reagent and solvent combinations in its top-1 recommendation for over half of the cases as listed in Table 1. Furthermore, if we expand our assessment to the top-10 recommended reaction contexts, the success rate of identifying at least one reaction condition for each reaction increases to about 73%, which highlights the capacity of the model to provide potentially relevant conditions for guiding experimentalists in chemical research.

**Table 1:** Top-k accuracy for identifying at least one ground truth reaction condition

	Top-1 (%)	Top-3 (%)	Top-10 (%)	Top-20 (%)
Exact matches	53.27	68.82	73.42	74.08

The performance of the model in predicting multiple reaction conditions for a given reaction is summarized in Table 2. For this assessment, we categorized the testing reactions into subsets based on the available number of condition records. As listed in Table 2, given the top-20 recommendations by the model, the success rate for predicting a single condition ranges from 67% to 90% across subsets. However, the model accuracy decreases when predicting second, third, and subsequent conditions, making it challenging to predict all conditions correctly. It is important to note that the dataset utilized in this work comprises a relatively modest proportion of reactions featuring multiple sets of conditions (8.8%). Therefore, a more diverse and comprehensive dataset could potentially enhance the model performance. Additionally, we observed instances where the contexts predicted by the model partially align with the recorded reaction conditions, introducing the possibility of valid substitutions. Nevertheless, as discussed above, our evaluation methodology defines a correct prediction with a strict criterion of an exact match to the reaction conditions in the data. This lack of partial match consideration contributes to diminished success rates, particularly as the number of condition records associated with a reaction increases. Further discussions on this point can be found in a subsection below.

**Table 2:** Top-20 accuracy in predicting multiple condition records for testing reactions

No. of records	No. of hit records				
	1	2	3	4	5
$1 (6067)^{1}$	70.74%	–	–	–	–
$2 (1097)^{1}$	89.88%	11.94%	–	–	–
3$$ (134)^{1}$$	80.60%	41.04%	4.48%	–	–
$4 (73)^{1}$	90.41%	60.27%	21.92%	4.11%	–
$5 (28)^{1}$	67.86%	53.57%	39.29%	21.43%	0%

^1^ The number in the parentheses represents the number of testing reactions

The performance of the model in predicting temperatures for reactions was evaluated using mean absolute error (MAE). The MAE for temperature prediction on the test set was $8.7^{\circ }{} {\textbf {C}}$. Predictions within $\pm10^{\circ }{\textbf {C}}$ and $\pm20^{\circ }{\textbf {C}}$ of the true values account for 71.1% and 88.6% of the test dataset, respectively. The mean ($44.6^{\circ }{\textbf {C}}$) and median ($20.0^{\circ }{\textbf {C}}$) of temperature distributions were used as baselines to assess prediction accuracy. Due to the wide range of reaction temperatures in the dataset (as shown in Additional file 1: Fig. S2), using the mean for prediction results in a MAE of $38.4^{\circ }{\textbf {C}}$, while using the median results in a MAE of $34.4^{\circ }{\textbf {C}}$. Therefore, relying solely on the average or median of the whole reaction dataset is not effective for synthesis planning. Conversely, the model temperature predictions offer chemists a reasonable estimate for reference.

### Assessment of recommended reaction conditions

**Fig. 6:**
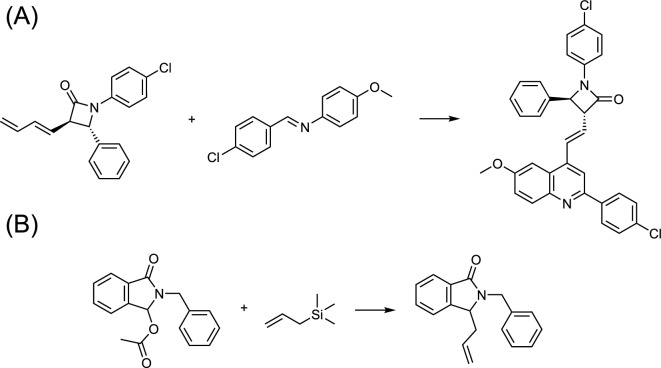
Reaction examples derived from the testing dataset: **A** aza-Diels-Alder reaction and **B** Friedel-Crafts alkylation

Figure 6 depicts two reaction examples from the testing set. The first example is an aza-Diels-Alder reaction (Fig. 6A), and the recorded and model-predicted reaction conditions are detailed in Tables 3 and 4, respectively. Table 4 shows that the recorded conditions were accurately predicted and ranked as the top recommendation. Interestingly, the second-ranked suggestion, involving $Y(OTf)_{3}$ as a reagent and acetonitrile as a solvent, resulted in a similar yield (87%) as the top-ranked condition in a study by Bhargava et al [62]. They also explored various Lewis acid catalysts with slightly lower yields (79–92%), such as zinc(II) chloride, indium(III) chloride, and scandium triflate. However, these alternative conditions were not included in the dataset because the Reaxys database typically retains only the highest yield condition from the literature, making it challenging to evaluate alternative conditions without reviewing the original publications individually.

In the second example reaction, a Friedel-Crafts alkylation (Fig. 6B), the model successfully predicted the two different reaction conditions in the dataset, as listed in Tables 5 and 6. Pin et al. [63] reported that the catalyst bismuth(III) trifluoromethanesulfonate was found to be effective and worked well in various solvents, including chloroform, tetrahydrofuran, nitromethane, and dichloromethane, with varying product yields. However, the dataset derived from Reaxys only had information on acetonitrile as the solvent, which limited the performance of the recommendation system. Nevertheless, the model accurately predicted dichloromethane as a viable alternative solvent, demonstrating its ability to provide valuable guidance to researchers beyond the dataset’s scope.

The two examples previously discussed highlight issues related to the format of documentation and the selection bias in choosing reaction conditions. Mercado et al. have emphasized the need to document detailed reaction information, such as the sequence of additives, concentrations of reactants, and reaction durations [64]. Moreover, it is important to note that yield information can be represented in various forms, including isolated yield, crude yield, conversion rates, and even as percentages of liquid chromatography area [52]. During data collection, it is essential that these yield metrics, along with detailed procedural information, are meticulously documented. In a notable development, the Open Reaction Database [65] has emerged as a leading data-sharing initiative, offering a repository of standardized chemical reactions. This open-source platform incorporates a review process that ensures the accuracy and integrity of its data sources. Such initiatives are important in overcoming obstacles in acquiring high-quality data to develop effective downstream machine learning models.

**Table 3:** Recorded reaction conditions for the aza-Diels-Alder reaction illustrated in Fig. 6A

Yield (%)	Reagent(s)	Solvent(s)	Temperature (°**C**)	Source
97	Magnesium bromide	Dichloromethane	20.0	Ref. [62]

**Table 4:** Predicted reaction conditions for the aza-Diels-Alder reaction illustrated in Fig. 6A

Rank	Reagent(s)	Solvent(s)	Temperature (°**C**)
1($\checkmark$)	Magnesium bromide	Dichloromethane	19.3
2	Yttrium(III) trifluoromethanesulfonate	Acetonitrile	21.9
3	Magnesium bromide	Acetonitrile; dichloromethane	18.5
4	Yttrium(III) trifluoromethanesulfonate	Dichloromethane	20.3
5	Magnesium bromide	Dichloromethane	21.0
6	Magnesium bromide	Acetonitrile	19.2
7	Magnesium bromide; yttrium(III) trifluoromethanesulfonate	Acetonitrile	22.3
8	Yttrium(III) trifluoromethanesulfonate	Acetonitrile; dichloromethane	20.4
9	Magnesium bromide; yttrium(III) trifluoromethanesulfonate	Acetonitrile; dichloromethane	21.0

**Table 5:** Recorded reaction conditions for the Friedel-Crafts alkylation illustrated in Fig. 6B

Yield (%)	Reagent(s)	Solvent(s)	Temperature (°**C**)	Source
99	Bismuth(III) trifluoromethanesulfonate	Acetonitrile	20.0	Ref. [63]
95	Silver trifluoromethanesulfonate	Acetonitrile	20.0	Ref. [66]

**Table 6:** Predicted reaction conditions for the Friedel-Crafts alkylation illustrated in Fig. 6B

Rank	Reagent(s)	Solvent(s)	Temperature (°**C**)
1($\checkmark$)	Silver trifluoromethanesulfonate	Acetonitrile	16.6
2	Trimethylsilyl trifluoromethanesulfonate	Dichloromethane	14.3
3	Silver trifluoromethanesulfonate	Dichloromethane	11.2
4	Trimethylsilyl trifluoromethanesulfonate	Acetonitrile	15.9
5($\checkmark$)	Bismuth(III) trifluoromethanesulfonate	Acetonitrile	19.4
6	Bismuth(III) trifluoromethanesulfonate	Dichloromethane	19.4
7	Trimethylsilyl trifluoromethanesulfonate	Acetonitrile; dichloromethane	13.6
8	Silver trifluoromethanesulfonate	Acetonitrile; dichloromethane	11.9
9	Bismuth(III) trifluoromethanesulfonate; trimethylsilyl trifluoromethanesulfonate	Dichloromethane	17.0
10	Bismuth(III) trifluoromethanesulfonate; silver trifluoromethanesulfonate	Dichloromethane	14.2

### Unsupervised learning reaction classification from reaction condition prediction

**Fig. 7:**
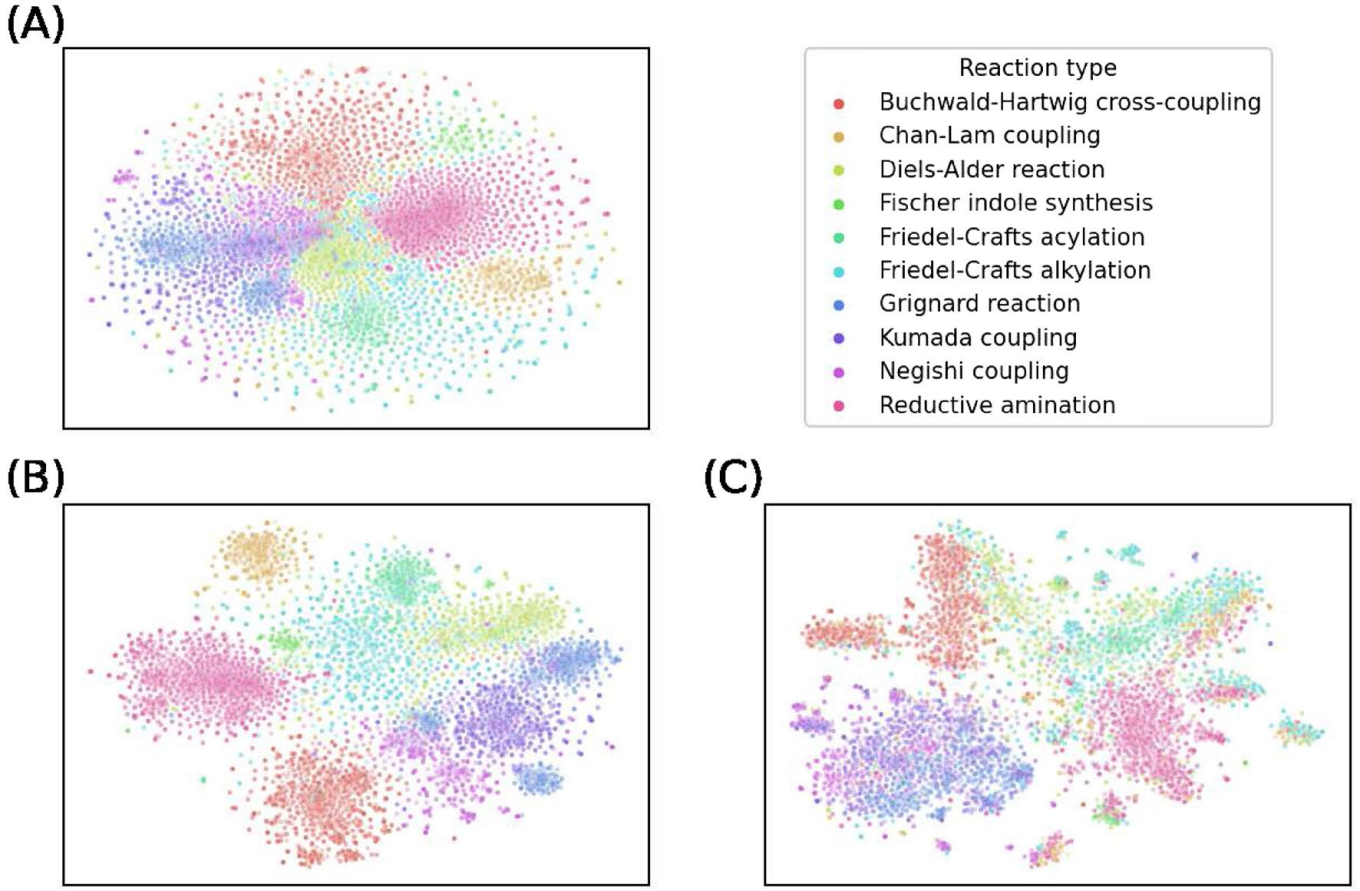
Embeddings of the testing data processed by the candidate generation model, extracted from **A** the shared layer, **B** the reagent task layer, and **C** the solvent task layer. Data points are color-coded based on their respective reaction types

Despite neural networks often being considered as black-box models, efforts have been made to enhance their interpretability [67, 68]. In this study, the focus was on the shared layer, reagent task layer, and solvent task layer of the candidate generation model (Fig. 2A). Testing data was passed through these layers, and the resulting embedding vectors were analyzed using t-SNE [69] for dimensionality reduction. As shown in Fig. 7, distinctive clustering patterns were observed in the shared layer embeddings, indicating the ability of the model to capture structural changes between reactants and products across different chemical reactions. In the reagent task layer, similar clustering patterns emerged, highlighting differences in reagents and catalysts used in different reaction types. However, the solvent layer embeddings showed partial overlap among some reactions. For example, reactions like Kumada coupling, Negishi coupling, and Grignard reaction were mixed together due to their common use of polar solvents such as diethyl ether and tetrahydrofuran [70–72]. Overall, the model’s predictions showed more overlapping tendencies in solvent selection compared to the reagent selection, which is consistent with established chemical intuition.

## Conclusions

This work introduces a novel approach to recommend reaction contexts, addressing the challenges of predicting appropriate reagents, solvents, and reaction temperatures for chemical reactions. The methodology involves a combination of a multi-label classification model and a ranking model to predict reaction conditions. To tackle the scarcity of unfavorable reaction contexts in the database, a concept of generating fictitious reaction conditions from the outcomes of the trained multi-label classification model was introduced. This augmentation strategy aids in refining the training process of the ranking model.

The proposed two-stage model was trained across ten reaction types, yielding an impressive 73% accuracy in exact top-10 matches for at least one condition set documented for each reaction in the test dataset. Additionally, the evaluation of the model demonstrates its ability to predict multiple suitable reaction conditions, with accuracy rates varying based on the number of condition records associated with each reaction. The success in suggesting alternative reaction conditions beyond the scope of the dataset highlights its potential to inspire innovative approaches in chemical research. Furthermore, the exploration of unsupervised learning using t-SNE embeddings provides valuable insights into the ability of the model to capture underlying chemical patterns. Clustering patterns observed among the shared, reagent, and solvent task layers demonstrate the capability of the model to differentiate between diverse chemical reactions and identify reagents and solvents specific to different reaction types.

We believe that this model can integrate with CASP. This model can adeptly suggest and prioritize diverse reaction conditions based on user-defined relevance scores. This functionality holds the potential to significantly enhance synthesis planning by uncovering more valuable and efficient retrosynthetic pathways, thereby advancing the field of chemical synthesis.

### Supplementary Information

**Additional file 1: Figure S1. **The label distribution of **A** reagents and **B** solvents after data reprocessing. Detailed names of reagents and solvents can be found in the data/reaxys_output/ label_processed directory. **Figure S2.** The distribution of temperatures in the reaction dataset used in this work. **Figure S3.** The distribution of yields in the reaction dataset used in this work. **Figure S4.** The distribution of reactions documented with varying numbers of conditionsin the dataset. **Figure S5.** The hyperparameter tuning results of the first candidate generation model. **Figure S6.** The hyperparameter tuning results of the second temperature prediction and ranking model. **Table S1.** Optimized hyperparameters for the first model. **Table S2.** Optimized hyperparameters for the second model.

## Data Availability

Full code and reaction IDs for searching the reactions are available at: https://github.com/Lung-Yi/rxn_yield_context.

## Abbreviations

CASP: Computer-aided synthesis planning
MAE: Mean average error
SMILES: Simplifed molecular input line entry specifcation
t-SNE: T-distributed stochastic neighbor embedding
