# Supplementary material for: Enhancing chemical synthesis: a two-stage deep neural network for predicting feasible reaction conditions
Source: J Cheminform. 2024 Jan 24;16:11. doi: 10.1186/s13321-024-00805-4 (PMC11301986; doi:10.1186/s13321-024-00805-4)
Supplement: Supplementary file 1 — Additional file 1: Figure S1. The label distribution of A reagents and B solvents after data reprocessing. Detailed names of reagents and solvents can be found in the data/reaxys_output/ label_processed directory. Figure S2. The distribution of temperatures in the reaction dataset used in this work. Figure S3. The distribution of yields in the reaction dataset used in this work. Figure S4. The distribution of reactions documented with varying numbers of conditionsin the dataset. Figure S5. The hyperparameter tuning results of the first candidate generation model. Figure S6. The hyperparameter tuning results of the second temperature prediction and ranking model. Table S1. Optimized hyperparameters for the first model. Table S2. Optimized hyperparameters for the second model. [file 13321_2024_805_MOESM1_ESM.pdf]

Enhancing Chemical Synthesis: A Two-Stage Deep Neural Network for Predicting Feasible Reaction Conditions

Lung-Yi Chen[a] and Yi-Pei Li[a][b]*

[a] Department of Chemical Engineering, National Taiwan University, No. 1, Sec. 4, Roosevelt Road, Taipei, 10617, Taiwan.

[b] Taiwan International Graduate Program on Sustainable Chemical Science and Technology (TIGP-SCST), No. 128, Sec. 2, Academia Road, Taipei, 11529, Taiwan.

*E-mail: yipeili@ntu.edu.tw

Supporting Information

Data Preparation and Code Availability

The protocols of data preprocessing, model training, and model evaluations are all included in the associated GitHub repository: `https://github.com/Lung-Yi/rxn_yield_context.git`. The trained models are provided in the path: `save_models/`. In this study, the reaction dataset retrieved from Reaxys[1] consists of 10 reaction types, including Buchwald–Hartwig cross coupling, Chan–Lam coupling, Diels–Alder, Fischer indole synthesis, Friedel–Crafts acylation, Friedel–Crafts alkylation, Grignard reaction, Kumada coupling, Negishi coupling, and reductive amination. The corresponding reaction IDs for searching the reactions are provided in the repository path: `data/reaxys_input/`.

Analysis of Reagent and Solvent Distributions

Chemical research often leans towards specific reagent and solvent categories due to factors like cost, availability, stability, and safety. This study followed suit by excluding rarely used labels and keeping only chemicals reported at least 10 times in the dataset. Following the data preprocessing steps outlined in the manuscript, the remaining dataset comprises 1,320 labels for the reagent class and 87 labels for the solvent class. Despite our best efforts, an imbalanced distribution persists, resulting in a 'long tail' data distribution, as illustrated in Figure S1.

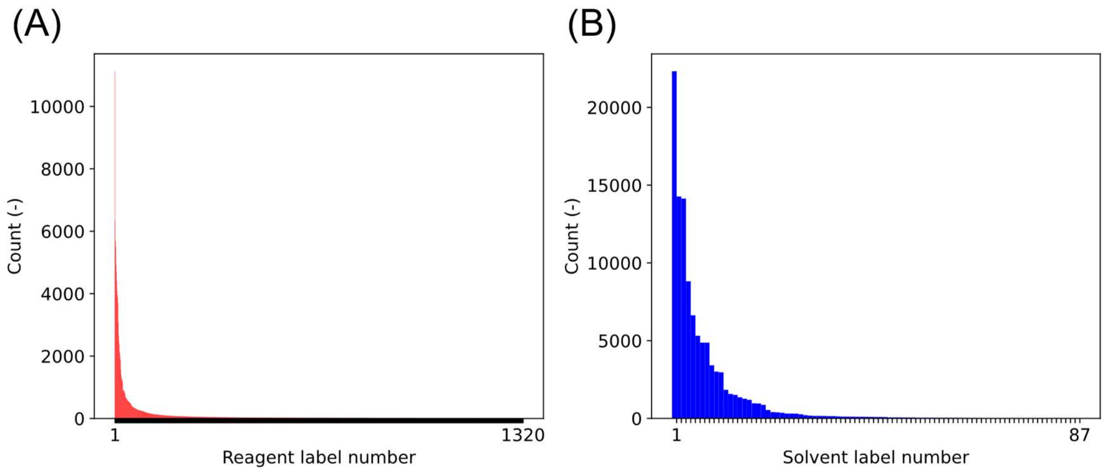

Figure S1. The label distribution of (A) reagents and (B) solvents after data reprocessing. Detailed names of reagents and solvents can be found in the `data/reaxys_output/label_processed` directory.

Examination of Reaction Temperatures and Yields

The distribution plot of reaction temperatures (Figure S2) reveals that some reactions occur at low-temperature ice bath conditions, while others take place at higher temperatures. Notably, a significant portion of chemical reactions happens at ambient temperature, approximately around 20 °C. In the distribution plot of reaction yields (Figure S3), it is evident that reactions with higher yields constitute a significant proportion of the dataset. This is partially attributed to Reaxys' tendency to include only the reaction condition with the highest reported yield while omitting conditions with lower yields from the same paper. This practice introduces bias into the yield distribution and restricts the diversity of recorded reaction conditions. As shown in Figure S4, the majority of chemical reaction records in the dataset provide only a single set of reaction conditions. In actual chemical synthesis literature, chemists frequently explore various combinations of catalysts and solvents to investigate yield variations.

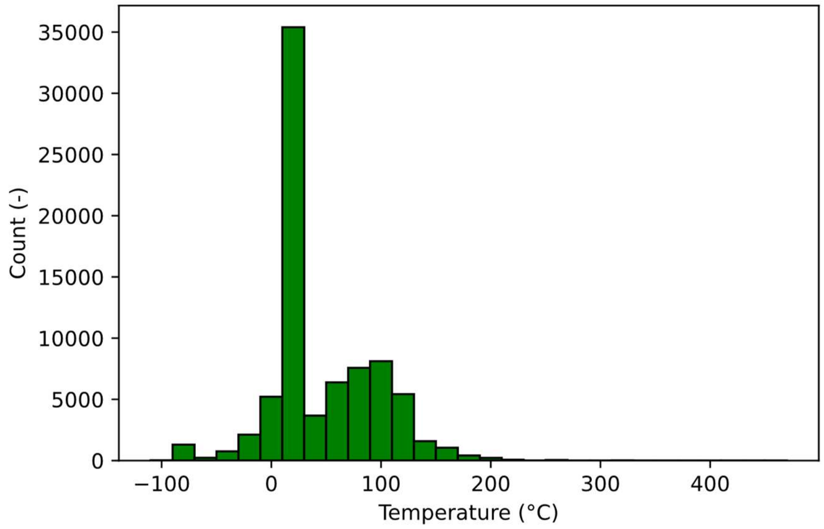

Figure S2. The distribution of temperatures in the reaction dataset used in this work.

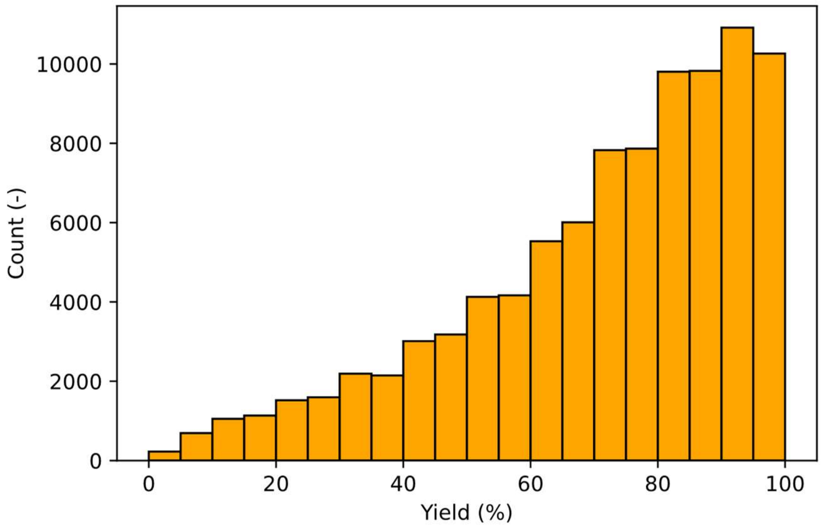

Figure S3. The distribution of yields in the reaction dataset used in this work.

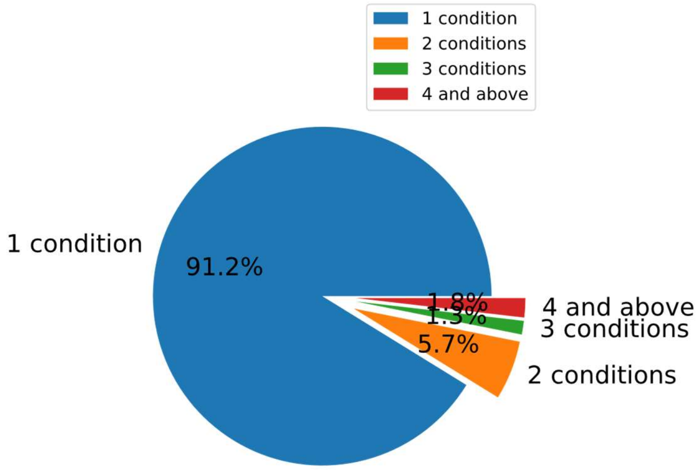

Figure S4. The distribution of reactions documented with varying numbers of conditions in the dataset.

Hyperparameter Tuning

In this study, the model hyperparameters were manually adjusted, and their validation performances were tracked using the wandb package,[2] as shown in Figures S5 and S6. For the first candidate generation model, which involved two multi-label prediction tasks, hyperparameter tuning aimed to maximize the summation of the f1-scores for reagent and solvent tasks, with both threshold values set to 0.3. In contrast, hyperparameters did not significantly impact temperature prediction accuracy for the second model, which was used for ranking and temperature prediction. Therefore, the second model's endpoint was set as the top-20 accuracy, which primarily assessed ranking performance. The optimized hyperparameters for the models can be found in Tables S1 and S2. Other hyperparameters are available in the Python file: rxn_yield_context/train_multilabel/args_train/args_rxn.py.

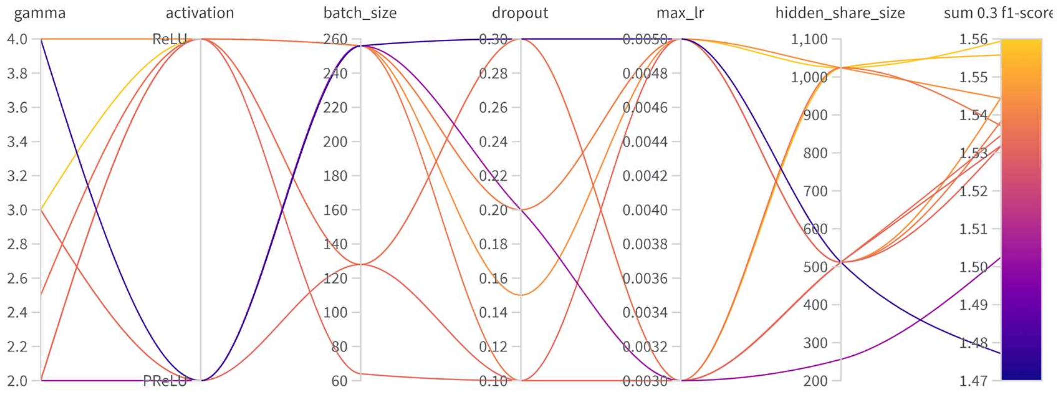

Figure S5. The hyperparameter tuning results of the first candidate generation model.

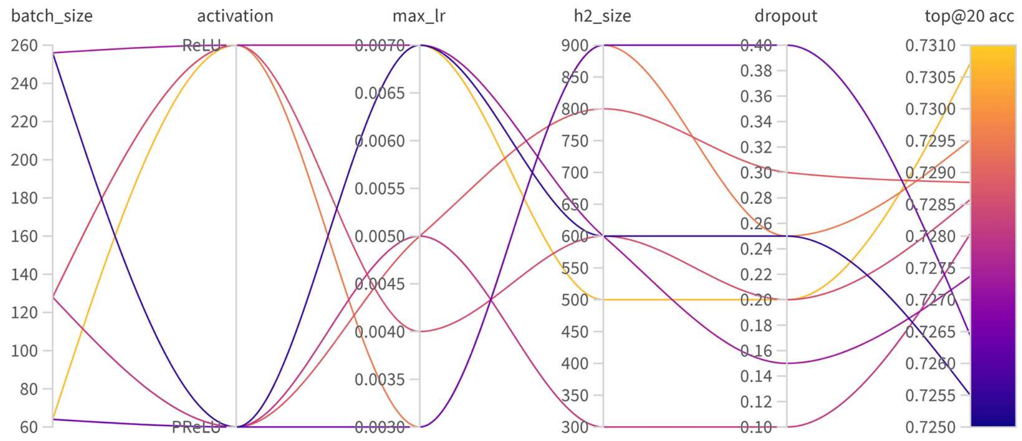

Figure S6. The hyperparameter tuning results of the second temperature prediction and ranking model.

Table S1. Optimized hyperparameters for the first model.

Parameter	Value	Description
activation	ReLU	activation function
batch_size	256	batch size
dropout	0.2	dropout probability in linear layer
gamma	3	modulating factor in focal loss
hidden_share_size	1024	dimensionality of hidden layers in shared layer
max_lr	5e-3	maximum learning rate

Table S2. Optimized hyperparameters for the second model.

Parameter	Value	Description
activation	ReLU	activation function
batch_size	64	batch size
dropout	0.2	dropout probability in linear layer
h2_size	500	dimensionality of hidden layers after reaction fingerprint
max_lr	7e-3	maximum learning rate

References

(1) *Reaxys*. https://www.reaxys.com/ (accessed January 07, 2023).

(2) *wandb*. https://github.com/wandb/wandb (accessed August 27, 2023).
